# The Effect of Metabolic Bariatric Surgery on Cardiovascular Risks: A Prospective Study Measuring Antibodies to Apolipoprotein A-1

**DOI:** 10.1007/s11695-024-07621-7

**Published:** 2025-01-03

**Authors:** Mohamed Osama Soliman Elgazawey, Sarah EL-Sayegh, Sameh Mikhail, Amr Mohamed AbdelFattah Ayad, Amir K. Abosayed

**Affiliations:** Cairo University Hospitals, Cairo, Egypt

**Keywords:** Metabolic bariatric surgery, Cardiovascular diseases, Anti-Apo A1 antibodies

## Abstract

**Background:**

Obesity is a chronic disease associated with other associated medical problems, including atherogenic dyslipidemia. Metabolic bariatric surgery (MBS) has been shown to reduce long-term cardiovascular risk (CVR). Anti-ApoA-1 antibodies (AAA1) are independently associated with cardiovascular disease, which remains a major cause of death in individuals with obesity. This study aimed to determine the effect of MBS on anti-ApoA-1 antibodies. We also looked for changes in lipid parameters, insulin resistance, inflammatory profile, and percentage of total weight loss (%TWL).

**Methods:**

We assessed 72 patients before surgery and 12 months postoperatively. Clinical history and measurements of body mass index (BMI), lipid profile (including non-HDL cholesterol, TG/HDL-C ratio, TG-Gly index, total cholesterol to HDL ratio), AAA1, CRP, fasting plasma glucose (FPG), HbA1c, and HOMA-IR were measured/calculated at each point.

**Results:**

MBS significantly improved BMI, %TWL, lipids, anti-ApoA-1 antibodies, CRP, HBA1c, FBG, and HOMA-IR. Baseline AAA1 antibodies were positive in 38.9% and were associated with higher CRP levels, total cholesterol, LDL-C, total cholesterol to HDL ratio, and non-HDL cholesterol. One year after MBS, there was a significant reduction in anti-ApoA-1 antibodies (*p* < 0.001). Furthermore, there was a significant postoperative correlation between anti-ApoA-1 antibodies with total cholesterol. Also, there were significant correlations between HBA1C (%), TG-Gly index, and HOMA-IR.

**Conclusions:**

Antibodies to apolipoprotein A-1 levels are significantly reduced following MBS. Furthermore, there was a notable improvement in the HBA1C, CRP, and lipid profile.

**Graphical Abstract:**

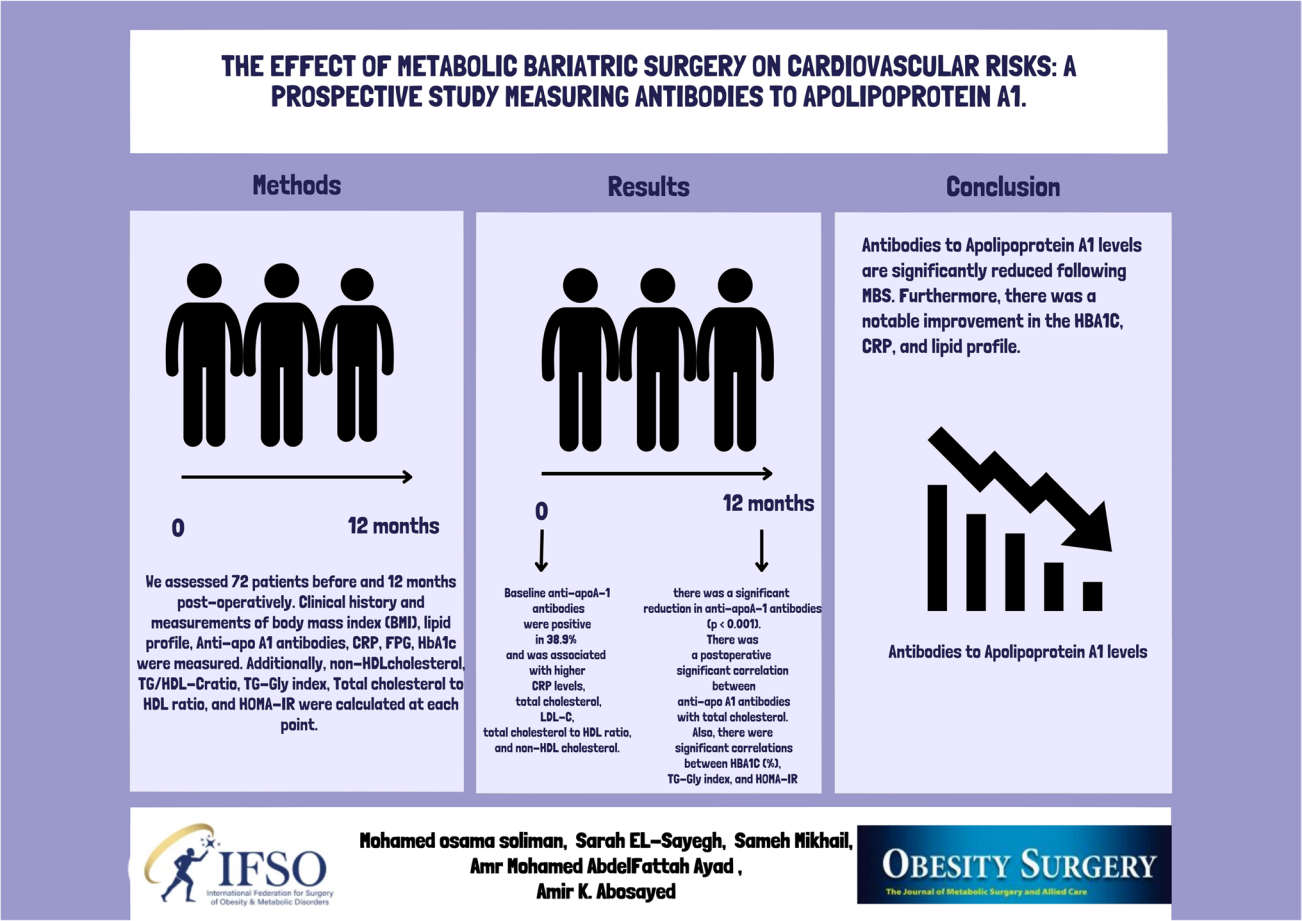

## Introduction

Obesity is an independent predictor of cardiovascular disease (CVD) and is correlated with a heightened risk of both morbidity and mortality. Despite growing global awareness, the prevalence of obesity remains unabatedly high [1]. Adipocyte hypertrophy in people with obesity initiates a series of interconnected pathophysiological mechanisms that culminate in endothelial dysfunction [2], oxidative stress, and lipoprotein modification [3]. It also leads to activation of the immune system and inflammatory pathway, as well as alterations in adipokine production [4].

The assessment of cardiovascular risk includes detailed clinical evaluation and laboratory investigations [5]. Clinical parameters such as blood pressure and medical history provide crucial insights into individual risk profiles. Laboratory tests include biomarkers like cholesterol levels and inflammatory markers and other calculated lab parameters such as triglyceride glucose (TyG) index [6], TG/HDL [7], and total cholesterol to HDL ratio [8].

Autoantibodies against the main protein component of high-density lipoprotein (HDL) and apolipoprotein A-1 (anti-ApoA-1 IgG) have become recognized as an independent biomarker for cardiovascular disease and death throughout the last 10 years [5, 6]. Through its interactions with immunological receptors, anti-apoA-1 IgG demonstrates pro-inflammatory features [7] and may also deleteriously influence HDL function [8]. It has been demonstrated to contribute to plaque instability and facilitate atherothrombosis [9, 10]. Additionally, it is linked to higher amounts of oxidized low-density lipoprotein (LDL), an essential element in all phases of atherosclerosis [11].

Anti-apoA-1 IgG was initially identified in patients with autoimmune disease and was linked to atherogenesis and adverse cardiovascular outcomes [12, 13]. Several studies further supported the link between anti-apoA-1 IgG and cardiovascular disease. Anti-apoA-1 IgG was established as an independent predictor of cardiovascular outcome following myocardial infarction [8]. Elevated autoantibody titers were found to be prevalent among patients with acute coronary syndrome [12]. Within the general population, anti-apoA-1 IgG is independently associated with CVD [10] and an independent predictor of all-cause mortality [9].

Metabolic bariatric surgery results in long-term weight loss and reduced CVD with its related morbidity and mortality [13, 14]. Some studies have found post-surgical reductions in inflammation and oxidative stress indicators and improvements in HDL structure and function [15–17].

## Patients and Methods

This prospective observational cohort study included 80 patients presenting to the bariatric outpatient clinic at Kasr Al Ainy hospitals from January 2022 to November 2023. Patients were scheduled for metabolic bariatric surgery if they were obese grade III or obese grade II with associated medical problems and fit for surgery under general anesthesia.

These patients fulfilled the institutional criteria for MBS eligibility. An independent surgeon, not involved in the study, discussed with the patients’ various surgical options. Laparoscopic sleeve gastrectomy and one anastomosis gastric bypass are the two most common procedures performed in our institution. After signing an informed consent for the chosen procedure, patients were offered to participate in our study.

### The Study Outcomes

The primary outcome of this study was the effect of MBS on the patients’ anti-ApoA-1 antibodies, and the secondary outcome was the correlation of anti-ApoA-1 antibodies with the patient’s other clinical, laboratory, and calculated parameters.

### Sample Collection and Biochemical Assay

Seven milliliters of blood was collected from each patient and divided as follows: 2 ml of blood was withdrawn into EDTA tube for measuring HbA1C and 2 ml of blood was withdrawn into a fluoride tube for measuring serum fasting glucose. Three milliliters of blood was withdrawn to a red-topped serum separator tube, serum was harvested by centrifugation, and samples were analyzed for serum fasting insulin, lipid profile, CRP, and anti-ApoA-1 antibodies. Clinical chemistry analysis was done on Dimension RxL Max (Siemens, USA), while serum insulin was assayed on cobas e 411 (Rosh Diagnostics, Germany). Detection of anti-ApoA-1 antibodies was done using commercially available ELISA assay kits supplied by Chongqing Biospes Co. (Catalog No. BZEK1825). The reference range, as stated by the manufacturer for this kit, is (12.29–21.33) µg/ml. The optical density (OD) was measured under 450-nm wavelength by Infinite F50 Plus (Tecan Group Ltd., Männedorf, Switzerland).

### Postoperative Management and Follow-Up

There were no major surgical events in the form of bleeding, leakage, and coagulative conditions among the study participants. All of the patients were discharged home on day 2 postoperative. Gradual diet progression was followed according to written instructions provided to the patients. Postoperative prophylaxis with low molecular weight heparin (LMWH) begins on day 1 and continuing through day 15 at a dosage of 1 mg/kg per day, with a maximum dose of 120 mg per day.

Patients’ follow-up visit, with the team conducting this study, was scheduled at 12 months postoperatively. The visit included a clinical examination, a weight assessment, and a laboratory assessment. The percentage of total weight loss (%TWL) was calculated as = [(preoperative weight − postoperative weight)/preoperative weight] × 100 [18].

### Statistical Methods

The patient’s data were analyzed by SPSS version 28. Data summarization was done by calculating mean, standard deviation, median, minimum, and maximum for quantitative variables and frequencies (number of cases) and relative frequencies (percentages) for categorical variables. An unpaired *t*-test was used to compare between groups in normally distributed quantitative variables, while a non-parametric Mann–Whitney *U* test was used for non-normally distributed quantitative variables. A paired *t*-test was used in normally distributed quantitative variables to compare serial measurements within each patient, while a non-parametric Wilcoxon signed-rank test was used for non-normally distributed quantitative variables. The chi-square (2) test was used in comparing categorical data. An exact test was performed when the expected frequency was less than 5. The Spearman correlation coefficient revealed correlations between quantitative variables. Results were considered statistically significant if the *p*-value was ≤ 0.05.

The sample size was calculated using “statistics and sample size pro” considering the following data: The mean (SD) preoperative anti-apoA-1 IgG median is 0.70 (0.07) vs. 0.53 (0.09) 6 months after bariatric surgery with alpha error 0.05 and the power of the study is 99%. The sample size should include 12 patients. Adding a potential of dropouts of 20%, the minimum sample size should be 15 patients. The researcher decided to recruit 80 patients for the sake of other outcomes.
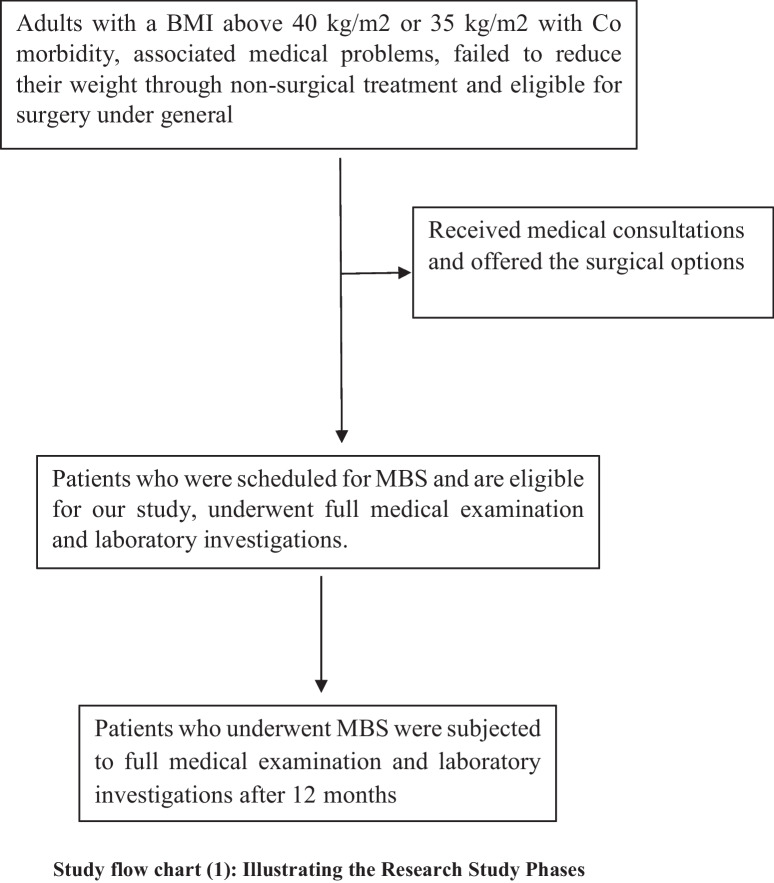


## Results

This study included initially 80 patients. Eight patients were lost during follow-up. It included the analysis of 72 patients. The mean age of the studied patients was 36.75 ± 10.32, and the majority were females (60 patients; 83.3%).

They had a mean weight of 137.80 ± 24.42 kg and a mean BMI of 50.66 ± 9.03 kg/m^2^. The associated medical problems were dyslipidemia, hypertension, and diabetes mellitus with a prevalence of 63.9%, 30.6%, and 38.9%, respectively (Table 1). The mean of preoperative systolic and diastolic blood pressure was 139.44 ± 17.59 and 94.86 ± 7.92, respectively.

**Table 1:** Baseline data of the study patients (72 patients)

Age		36.75 ± 10.32	
Weight		140.17 ± 24.87 kg	
BMI		51.82 ± 9.18	
Waist circumference		112.86 ± 25.65	
		Count	%
Gender	Female	60	83.3%
	Male	12	16.7%
Associated medical problems	Dyslipidemia	46	63.9%
	Diabetes mellitus	28	38.9%
	Hypertension	22	30.6%
Anti-Apo-A-1 abs positivity		28	38.9%

Preoperative laboratory analysis revealed that the mean of the HbA1C was 6.47 ± 1.74%, while fasting plasma glucose ranged from 67 to 330 mg/dl with a median of 120.50 mg/dl, and fasting insulin ranged from 3.27 to 60.73 µIU/ml with a median 11.39 µIU/ml. Regarding HOMA-IR, it ranged from 0.57 to 27.53, with a median of 3.37.

The mean value of total cholesterol was 199.61 ± 45.18 mg/dl, HDL-C was 44.14 ± 10.72 mg/dl, and LDL-C was 131.19 ± 47.07 mg/dl. While triglyceride levels ranged from 64 to 307 mg/dl, with a median value of 121. CRP ranged from 1 mg/l to 25 mg/l with a median value of 10.

The calculated triglycerides-glycemic (TG-Gly) index was 4.86 ± 0.24. The triglycerides/HDL (TG/HDL) ratio ranged from 0.92 to 9.30 with a median value of 3, and the total cholesterol to HDL ratio ranged from 1.93 to 9.33 with a mean value of 4.67.

Preoperatively, 28 participants (38.9%) were positive for anti-ApoA-1 antibodies with a mean value of 21.42 ± 3.72 µg/ml.

The postoperative follow-up data are shown in Table 2; there was an overall significant statistical postoperative reduction in clinical and laboratory parameters (*P* value < 0.001).

**Table 2:** The preoperative and postoperative data of the study patients

	Preoperative	12 months postoperative	*P-value
Weight (kg)	140.17 ± 24.87	86.86 ± 14.21	< 0.001
BMI (kg/m^2^)	51.82 ± 9.18	32.09 ± 5.24	< 0.001
Waist circumference(cm)	112.86 ± 25.65	85.06 ± 20.39	< 0.001
Systolic BP (mm Hg)	139.44 ± 17.59	116.39 ± 8.93	< 0.001
Diastolic BP (mm Hg)	94.86 ± 7.92	81.39 ± 7.93	< 0.001
HBA1C (%)	6.47 ± 1.74	4.89 ± 0.62	< 0.001
Fasting glucose (mg/dl)	136.92 ± 54.12	88.1 ± 12.4	< 0.001
Fasting insulin	11.39 (3.27–60.73) **	2.25 (0.37–9.70) **	< 0.001
HOMA-IR	3.37 (0.57–27.53)**	0.45 (0.1–2.2)**	< 0.001
Total cholesterol (mg/dl)	199.61 ± 45.18	161.58 ± 38.39	< 0.001
Triglycerides (mg/dl)	121 (64–307)**	87 ( 48–269)**	< 0.001
HDL-C (mg/dl)	44.14 ± 10.72	58.06 ± 12.7	< 0.001
LDL-C (mg/dl)	131.19 ± 47.07	88.83 ± 35.52	< 0.001
(TG-Gly) index	4.86 ± 0.24	4.44 ± 0.27	< 0.001
TG/HDL	3 (0.92–9.3)	1.61 (0.63–5.85)	< 0.001
Total cholesterol to HDL ratio	4.79 ± 1.68	2.94 ± 1.01	< 0.001
CRP (mg/l)	10(1–25)**	1 (0.4–6)**	< 0.001
Anti-Apo A1 antibodies levels (µg/ml)	21.42 ± 3.72	15.62 ± 4.58	< 0.001

*P value is significant < 0.05

**Data represented by median and interquartile ranges

**Fig. 1:**
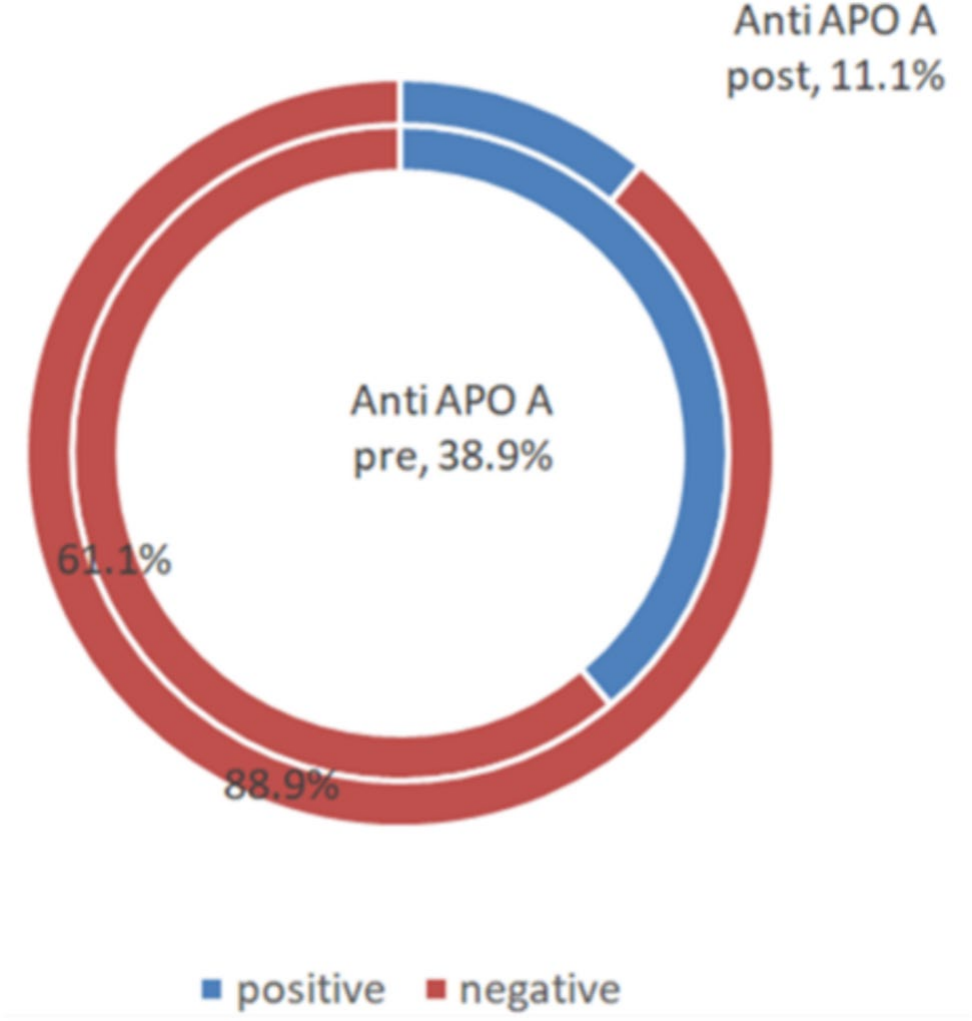
The difference in Apo A1 positivity pre- and postoperative

There was a reduction in the number of patients with positive anti-ApoA-1 antibodies (eight patients, 11.1%) as shown in Fig. 1.

We demonstrated that there were significant correlations between HOMA-IR and TG-Gly index preoperatively and postoperatively (*r* = 0.387 and 0.347, *P*-value = 0.001 and 0.003), respectively. However, there was a correlation between preoperative HbA1C and HOMA-IR (*r* = 0.470, P-value =  < 0.001), While there is no postoperative correlation between HbA1C and HOMA-IR (*r* = 0.148, *P*-value = 0.216).

**Fig. 2:**
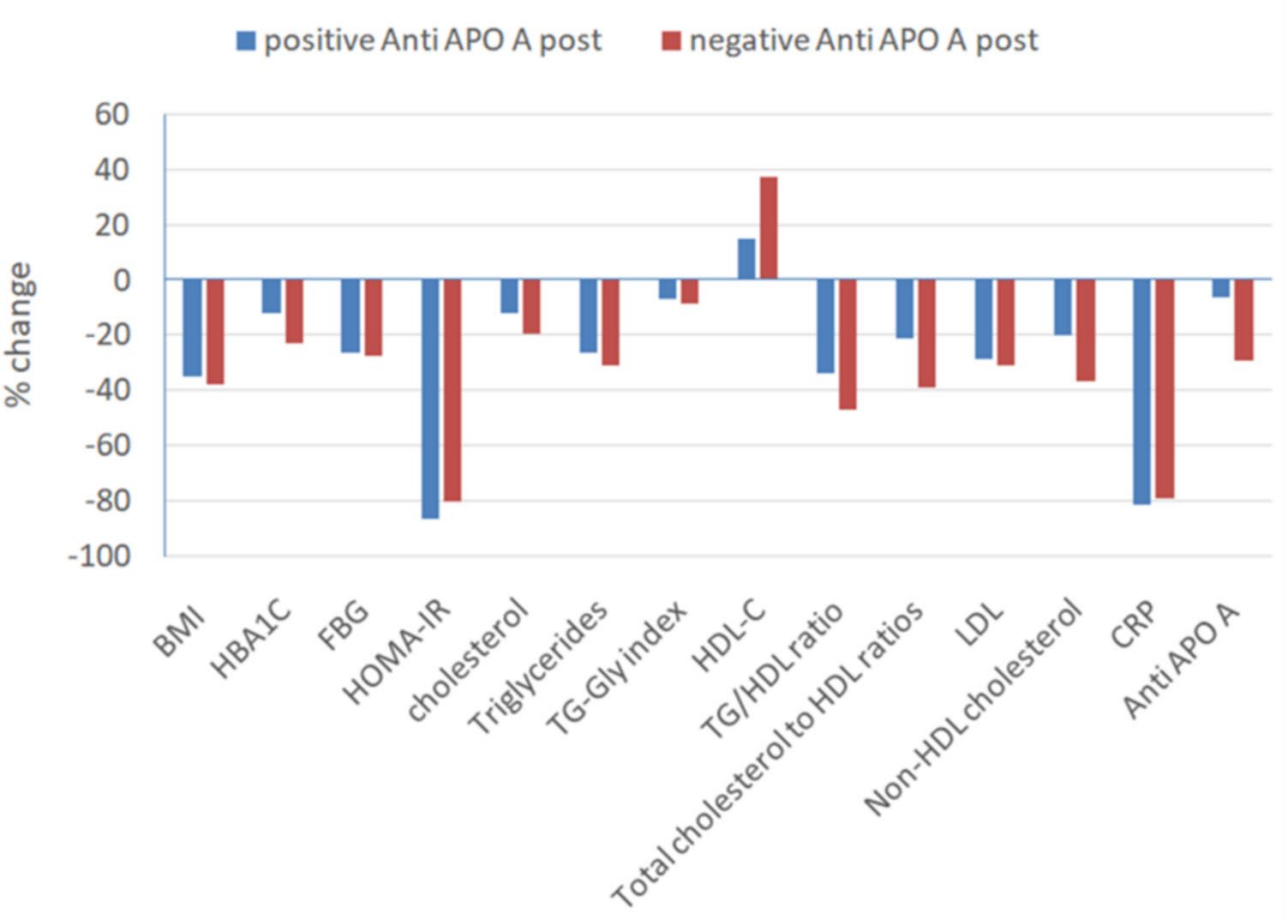
Difference between seropositive and seronegative patients postoperatively

Figure 2 shows the difference in postoperative parameters between anti-ApoA-1 antibodies seropositive and anti-ApoA-1 antibodies seronegative patients.

Comparing patients with anti-ApoA-1 antibodies seropositive to anti-ApoA-1 antibodies seronegative patients revealed, as shown in Table 3, a statistically significant difference in preoperative patient’s age, weight, waist circumference, LDL, CRP, and anti-ApoA-1 antibodies (*P*-value = 0.017, 0.019, 0.035, 0.023, < 0.001, < 0.001), respectively, and also revealed a statistically significant difference 12-month postoperative systolic blood pressure, total cholesterol, and anti-ApoA-1 antibodies (*P*-value = 0.003, 0.024, < 0.001), respectively.

**Table 3:** Comparing patients with anti-ApoA-1 Antibodies seropositive to anti-ApoA-1 antibodies seronegative patients

	Preoperative			12 months postoperative		
	Anti-ApoA-1 antibodies seropositive (*n* = 28)(38.8%)	Anti-ApoA-1 antibodies seronegative	*P*-value	Anti-ApoA-1 antibodies seropositive (*n* = 8) (11.1%)	Anti-ApoA-1 antibodies seronegative	*P*-value
Age (years)	33.14 ± 9.18	39.05 ± 10.44	0.017*	30.75 ± 8.4	37.5 ± 10.35	0.081
Weight (kg)	131.64 ± 19.38	145.59 ± 26.61	0.019*	81.00 ± 16.09	87.59 ± 13.92	0.218
BMI (kg/m^2^)	49.70 ± 7	53.17 ± 10.18	0.091	32.08 ± 4.38	32.09 ± 5.37	0.995
Waist circumference (cm)	104.93 ± 25.75	117.91 ± 24.56	0.035*	80.25 ± 20.06	85.66 ± 20.51	0.484
Systolic BP (mm Hg)	136.43 ± 9.11	141.36 ± 21.2	0.179	125 ± 9.26	115.31 ± 8.35	0.003*
Diastolic BP (mm Hg)	92.86 ± 4.99	96.14 ± 9.14	0.054	77.5 ± 8.86	81.88 ± 7.74	0.142
HBA1C (%)	5.97 ± 1.1	6.79 ± 2	0.051	4.72 ± 0.45	4.91 ± 0.64	0.435
Fasting glucose (mg/dl)	126.79 ± 38.45	143.36 ± 61.63	0.207	82.75 ± 9.60	90.66 ± 13.14	0.105
Fasting insulin	11.15 ** (3.27–28.8)	11.39** (3.39–60.73)	0.817	2.53** (0.37–5.56)	2.25** (0.62–0.7)	0.473
HOMA-IR	3.48** (0.57–12.89)	3.28** (0.75–27.53)	0.277	0.60** (0.10–0.90)	0.45** (0.1–2.2)	0.563
Total cholesterol (mg/dl)	210.86 ± 36.61	192.45 ± 48.92	0.092	176 ± 13.09	159.78 ± 40.15	0.024*
Triglycerides (mg/dl)	132.07 ± 55.7	145.32 ± 55.3	0.488	82.25 ± 25.62	97.34 ± 45.14	0.430
HDL-C (mg/dl)	44.57 ± 11.11	43.86 ± 10.53	0.787	54.25 ± 14.84	58.53 ± 12.46	0.372
LDL-C (mg/dl)	149.29 ± 56.45	119.68 ± 36.17	0.023*	93.5 ± 27.02	88.25 ± 36.57	0.473
(TG-Gly) index	4.8 ± 0.23	4.9 ± 0.24	0.077	4.39 ± 0.17	4.45 ± 0.28	0.554
TG/HDL	3.27 ± 1.8	3.59 ± 1.88	0.460	1.78** (0.66–2.65)	1.61** (0.63–5.85)	0.943
Total cholesterol to HDL ratio	3.15 ± 0.82	2.81 ± 1.11	0.092	3.42 ± 0.75	2.88 ± 1.03	0.115
CRP (mg/l)	16.54 ± 3.56	6.95 ± 2.92	< 0.001*	1.75** (1–6)	1** (0.4–6)	0.050
Anti-ApoA-1 antibodies levels (µg/ml)	25.21 ± 2.94	19.01 ± 1.43	< 0.001*	23.6 ± 1.68	14.62 ± 3.77	< 0.001*
%TWL				35.4 ± 6.96	38.38 ± 5.96	0.474

*P value is significant < 0.05

**Data represented by median and interquartile ranges

Concerning %TWL for patients 12 months postoperatively, there is no statistical significance between postoperative Anti-ApoA-1 antibody seropositive and seronegative (mean = 35.43 ± 6.45, 37.97 ± 6.16, *P*-value = 0.277).

As shown in Table 4, univariate correlation was done to detect the strength and direction of relationships between each variable with anti-ApoA-1 antibodies. At baseline, there was a significant correlation between anti-ApoA-1 antibodies with total cholesterol, LDL-C, total cholesterol to HDL ratios, non-HDL cholesterol, and CRP. While postoperative data showed that there was a significant correlation between anti-ApoA-1 antibodies with total cholesterol, there were no statistically significant correlations between anti-ApoA-1 antibody levels and other variables seen in both groups at baseline and 12 months postoperatively.

**Table 4:** Correlation coefficients between anti-ApoA-1 IgG and other variables at baseline and 12 months post-operatively

	Baseline variables vs. anti-ApoA-1 IgG	12 months postoperative variables vs. anti-ApoA-1 IgG
BMI	*r* = − 0.068 *p* = 0.568	*r* = 0.028 *p* = 0.818
Systolic BP	*r* = 0.201 *p* = 0.091	*r* = 0.213 *p* = 0.072
Diastolic BP	*r* = 0.044 *p* = 0.711	*r* = − 0.229 *p* = 0.053
Total cholesterol	***r* = 0.316** *p* = 0.007*	***r* = 0.274** *p* = 0.020*
Triglycerides	*r* = 0.137 *p* = 0.251	r = − 0.029 *p* = 0.808
TG-Gly index	*r* = − 0.114 *p* = 0.342	*r* = − 0.071 *p* = 0.553
HDL-C	*r* = 0.031 *p* = 0.797	*r* = − 0.229 *p* = 0.053
LDL-C	***r* = 0.3** *p* = 0.011*	*r* = 0.202 *p* = 0.089
Total cholesterol to HDL ratio	***r* = 0.234** *p* = 0.048*	*r* = 0.162 *p* = 0.175
TG/HDL ratio	*r* = 0.081 *p* = 0.499	*r* = 0.008 *p* = 0.949
Non-HDL cholesterol	***r* = 0.295** *p* = 0.012*	*r* = 0.206 *p* = 0.082
HOMA-IR	*r* = − 0.139 *p* = 0.243	*r* = 0.066 *p* = 0.582
CRP	***r* = 0.646** *p* = < 0.001*	*r* = 0.232 *p* = 0.050

Spearman’s correlation coefficient between different variables at different time points. *Anti-apoA-1 IgG* antibodies to apolipoprotein A-1, *HDL-C* high-density lipoprotein cholesterol, *LDL-C* low-density lipoprotein cholesterol, *CRP* C-reactive protein, *HOMA-IR* homeostatic model of assessment of insulin resistance, *BMI* body mass index

Table 5 shows that the % change in anti-ApoA-1 antibodies was correlated with change in other parameters. It showed a significant relation between triglycerides % change and TG/HDL ratio % change. Then, multivariate linear regression model was done in Tables 6 and 7 to adjust for possible confounders as operation type which showed no significant correlations in Tables 6 and 7.

**Table 5:** The % change in anti-ApoA-1 antibodies is correlated with change in other parameters

BMI % change	Anti-ApoA-1 antibodies % change	
	Correlation coefficient	*P* value
	− 0.128	0.284
**HBA1C % change**	0.153	0.199
**FBG % change**	0.120	0.313
**HOMA-IR % change**	− 0.123	0.304
**cholesterol % change**	0.184	0.122
**Triglycerides % change**	**0.340**	0.003*
**TG-Gly index % change**	0.200	0.092
**HDL-C % change**	0.086	0.475
**TG/HDL ratio % change**	**0.255**	0.030*
**Total cholesterol to HDL ratios % change**	0.082	0.494
**LDL % change**	− 0.041	0.731
**Non-HDL cholesterol % change**	0.118	0.322
**CRP % change**	− 0.021	0.859
**SBP % change**	0.079	0.512
**DBP % change**	− 0.066	0.584

*P value is significant < 0.05

**Table 6:** A multivariate linear regression model to detect independent predictors of triglycerides % change

Model		Standardized coefficients beta	*P*-value
**Triglycerides % change**	**Anti-ApoA-1 antibodies % change**	0.277	0.021*
	**Smoking**	− 0.202	0.140
	**DM**	0.030	0.810
	**HTN**	− 0.162	0.214
	**Operation**	0.112	0.357
	**Age**	0.101	0.455

*P value is significant < 0.05

**Table 7:** A multivariate linear regression model to detect independent predictors of TG/HDL ratio % change

Model		Standardized coefficients beta	*P*-value
**TG/HDL ratio %**	**Anti-ApoA-1 antibodies % change**	0.188	0.132
	**Smoking**	− 0.125	0.382
	**DM**	0.003	0.980
	**HTN**	− 0.036	0.791
	**Operation**	− 0.135	0.291
	**Age**	− 0.032	0.821

In Table 6, a multivariate linear regression model was done to detect independent predictors of triglycerides % change. After adjustment for possible confounders, there was a significant positive correlation between the % change in anti-ApoA-1 antibodies and the % change in triglycerides, while no significance with the other confounders.

Moreover, a multivariate linear regression model was done in Table 7 to detect independent predictors of TG/HDL ratio % change. However, after adjustment for possible confounders, there was no significant correlation between the % change in anti-ApoA-1 antibodies and the % change in TG/HDL ratio %.

## Discussion

Anti-ApoA-1 IgG has emerged as a noteworthy biomarker associated with cardiovascular disease (CVD) and mortality [19]. Several studies stated that anti-ApoA-1 autoantibodies may be present in the general population but in low titers (up to 6.5%) and may be related to the vascular and immune aging processes [16].

Our study focused on a cohort of Egyptian obese patients to investigate the impact of metabolic bariatric surgery on the follow-up 12 months postoperatively on anti-apolipoprotein A-1 (anti-ApoA-1 IgG) auto-antibody titers and associated changes in lipid parameters.

Based on our findings, there was a reduction in the number of seropositive patients from 28 (38.9%) preoperatively to 8 (11.1%) postoperatively. Moreover, there was an overall significant statistical postoperative reduction in anti-apolipoprotein A-1 antibody levels. This trend aligned with the results of a study conducted by Adam et al. [20] and Adam et al. [21]. However, the prevalence of autoantibody positivity status at baseline in our study (38.9%) was greater than that conducted by Adam et al. (2022), which was 25%.

The study showed that the individuals with positive autoantibody status exhibited significantly lower baseline body weight and were younger. Notably, our findings indicate a higher %TWL in patients who tested negative for autoantibodies postoperatively compared to those who tested positive, although it was not statistically significant.

Also, patients who were anti-ApoA-1 antibody seropositive showed a significant preoperative increase in LDL-C (*P*-value = 0.023) and a postoperative increase in systolic blood pressure and total cholesterol (*P*-value = 0.003, 0.024), respectively. These findings suggested a higher cardiovascular risk in individuals with positive autoantibody status.

Interestingly, in the current study, CRP levels were higher in patients whose antibody status was positive (16.54 ± 3.56) compared to negative (6.95 ± 2.92) with preoperative statistical significance (*P*-value =  < 0.001). However, postoperative CRP levels showed no statistical significance (*P*-value = 0.050).

CRP has been used as a biomarker of cardiovascular risk. At CVD risk, individuals can be classified according to the level of CRP into three groups: (1) low risk when CRP levels are below 1 mg/L, moderate risk when CRP levels are between 1 and 3 mg/L, and high risk when CRP levels exceed 3 mg/L [22, 23].

Several studies investigated the impact of MBS on cardiovascular risk by examining various cardiovascular biomarkers. It is known that obesity alters HDL metabolism, affecting ApoA-1 [24], and this could explain the presence of anti-ApoA-1 antibodies and therefore increased CVD risk.

In the present study, we assumed that MBS was responsible for the reduction in anti-ApoA-1 antibody levels with significant improvements in blood pressure, BMI, insulin resistance, hyperglycemia, inflammation, and the lipid profile, which supported the idea that MBS induces positive changes in cardiovascular biomarkers, resulting in a healthier lipid profile and reduced cardiovascular risk, as demonstrated in the studies by Adam et al. (21), Gómez et al. [24], Farias et al. [25], Doumouras et al. [26], and Adam et al. [21].

We demonstrated, at baseline, a positive correlation between anti-ApoA-1 IgG levels on one side and total cholesterol, LDL-C, total cholesterol to HDL ratios, non-HDL cholesterol, and CRP on the other side. However, there was a positive correlation between anti-ApoA-1 antibodies and total cholesterol.

A study by Bridge et al. [27] determined that the correlation analysis showed an inverse relationship between anti-ApoA-1 autoantibody responses and total cholesterol concentration (*r* =  − 0.32; *p* = 0.005) in HCV patients, in contrast to ours, which showed a positive correlation between anti-ApoA-1 IgG levels on one side and total cholesterol pre- and postoperatively. Moreover, the study agreed with our study that there is no correlation between anti-ApoA-1 antibodies and TG/HDL-C. However, in contrast to ours, it showed no correlation between total cholesterol to HDL ratios and anti-ApoA-1 antibodies.

Our results showed partial agreement with Adam et al. [18], which stated no correlation was found between anti-ApoA-1 IgG and lipid profile. Another study by Antiochos et al. [10] showed an inverse correlation between anti-ApoA-1 IgG and HDL-C; however, we showed no correlation between them.

Our study demonstrated a positive correlation with the % change before and after surgery between anti-ApoA-1 antibodies and triglycerides, while no significant correlation was observed with % change between anti-ApoA-1 antibodies and other parameters such as BMI, systolic and diastolic blood pressure, total cholesterol, HDL-C, LDL-C, TG-Gly index, TG/HDL ratio, total cholesterol to HDL, HBA1C, fasting glucose, HOMA-IR, and CRP.

These results demonstrated an association between anti-ApoA-1 autoantibodies and cardiovascular risk, and these are consistent with El-Lebedy et al. [16], who demonstrated this association in diabetic patients.

It is worth noting that the current study demonstrated a positive correlation between HOMA-IR and TG-Gly index preoperatively and postoperatively. In accordance with these findings, a study by Liu et al. [28] showed an elevation of the TyG index with a more severe IR, and a study by Hegab et al. [29] demonstrated a significant association between the TG-Gly index and HOMA-IR and might be applied as eligible indices of IR among overweight and/or obese Egyptians.

## Strengths and Limitations

The present study is one of a few prospective studies worldwide assessing the impact of MBS on anti-ApoA-1 antibodies, and as per our knowledge, it is the first study on the Egyptian population. However, the study is limited by the short-term follow-up of the study patients. Our sample’s higher proportion of women hindered our ability to explore sex-specific differences. More variables could have been studied to allow the development of a predictive model or confounding factors. These limitations highlight the need for larger and more diverse cohorts to better understand the complex dynamics of anti-ApoA-1 antibodies and their alterations post-MBS.

## Conclusion

In conclusion, our data show that metabolic bariatric surgery (MBS) reduces anti-ApoA-1 antibody levels and changes positivity status. Also, there was a significant improvement in lipid profile, HBA1C, and CRP. Further studies are needed to determine whether this contributes to the decrease in CVD risk observed in longitudinal cohort studies in patients after MBS. Significant correlations existed between HOMA-IR, TG-Gly index, and HBA1C (%). There was a preoperatively significant correlation between anti-ApoA-1 antibodies and total cholesterol, LDL-C, total cholesterol to HDL ratios, non-HDL cholesterol, and CRP, and a postoperatively significant correlation between anti-ApoA-1 antibodies and total cholesterol. Moreover, there were no statistically significant correlations between anti-ApoA-1 antibody levels and other variables seen in both groups at baseline and 12 months postoperatively.

## Data Availability

No datasets were generated or analysed during the current study.
